# Non-verbal cues in eyewitness testimonies do not predict accuracy or credibility assessments

**DOI:** 10.1038/s41598-025-89825-0

**Published:** 2025-02-12

**Authors:** Arman Raver, Torun Lindholm, Charlotte Alm

**Affiliations:** Department of Psychology, Stockholm University, Stockholm, Sweden

**Keywords:** Non-verbal cues, Eyewitness testimony, Credibility assessment, Memory accuracy, Native vs. non-native speakers, Psychology, Human behaviour

## Abstract

Non-verbal behaviour, such as facial expressions and body language, plays a critical role in assessments of witnesses’ credibility that inform legal decisions in cases involving crime. While prior research has primarily focused on associations between non-verbal cues and deception, this study investigates the relation between non-verbal cues and recall memory accuracy in honestly reported mock eyewitness testimonies. Using a sample of 36 video-recorded eyewitness testimonies about a violent crime (n = 680 statements), we examined whether non-verbal cues were associated with statement accuracy (correct vs. incorrect), witness credibility as rated by independent observers, and witnesses’ self-reported confidence. Additionally, we explored whether these associations differed for native vs. non-native speaking witnesses. Results revealed no associations between non-verbal cues and statement accuracy or perceived credibility. Furthermore, while non-native speakers were perceived as less credible, these perceptions were not related to non-verbal cues. Our findings contradict common beliefs by showing that non-verbal behaviour is not reliably related to accuracy or perceived credibility in eyewitness testimonies, highlighting the need for caution in their use in high-stakes legal contexts.

## Introduction

Non-verbal behaviour, such as facial expressions, gestures, body language, and gaze direction, influences how credible people are perceived in both everyday interactions and high-stakes legal contexts, specifically when jurors and the police assess witness credibility to inform legal decisions^1,2^. Prior research has primarily focused on how non-verbal behaviour is associated with deception, but effects have been weak and small^3–5^. However, many eyewitnesses in legal processes have no intent to deceive when giving their statement, but rather to describe an event as they actually recall it. Despite the prevalence of such honest testimonies and the belief in the diagnostic value of non-verbal cues for legal decision-making, no previous empirical study, to our knowledge, has examined the relation between non-verbal behaviour and recall memory performance. The present study explores whether non-verbal cues in eyewitness statements relate to recall memory accuracy as well as to perceived credibility. Moreover, we investigate whether any such relationship differs depending on whether eyewitnesses are testifying in their native- or a non-native language. This study expands on Raver et al.^6^ who showed that non-native, as compared to native speaking witnesses, were judged as less credible by independent observers, despite both groups providing equally accurate testimonies. Our study thus also addresses potential biases that can lead to unfair judgments of eyewitnesses, particularly non-native speakers.

### Non-verbal behaviours, testimony accuracy and perceived credibility

Non-verbal cues complement verbal communication, shaping the observer’s understanding and enhancing overall communication efficiency^7–9^. Swerts and Krahmer^10^ demonstrated that non-verbal cues, such as changes in facial expressions, can indicate a speaker’s certainty, referred to as Feeling of Knowing (FOK), in a given statement. These cues also affect how confident/uncertain the speaker appears to an observer. The role of non-verbal cues in communication becomes particularly critical in evaluations of eyewitness statements in the legal context. Prior research demonstrates that a witness’ non-verbal behaviour plays an important, yet often unreliable role in evaluations of the witness’ accuracy. For example, incongruence between the content of the testimony and the eyewitness’ behaviour, can negatively influence observers’ credibility judgments, potentially leading to incorrect conclusions^11–13^. Thus, if a witness provides detailed and accurate information but displays behaviours commonly associated with low confidence, such as fidgeting or avoiding eye contact, their testimony may be unfairly viewed as dishonest.

Furthermore, Lindholm^14^ found that differences in observed non-verbal cues, such as facial expressions (e.g. eyebrow movement, smile) and body movements (e.g. change of position, gestures), between native and non-native witnesses influenced how their credibility was assessed. Specifically, highly accurate native witnesses exhibited fewer facial expressions and were rated more credible than low-accurate native witnesses. In contrast, highly accurate non-native witnesses displayed more such facial expressions and were given equally low credibility ratings as low-accurate non-native witnesses^14^. Overall, these findings indicate that non-verbal cues, which do not always reflect actual accuracy, may lead to biased and unjust credibility judgments – especially against non-native eyewitnesses.

### Misconceptions among legal professionals about eyewitnesses’ non-verbal behaviours

When assessing witness credibility, jurors seem to consider both the content of the testimony and the manner of its delivery^1,15^. For example, Chalmers et al.^15^ observed that jurors often relied on witnesses’ nervousness and gaze direction to assess credibility, based on the belief that these cues reliably indicate dishonesty. Similarly, Denault et al.^1^ showed that judges relied on non-verbal cues, such as gestures, gaze direction, and body posture, when judging witness credibility, despite the lack of empirical evidence of the reliability of these cues. Moreover, judges disagreed on how non-verbal cues relates to accuracy, with some believing that incongruency (e.g. a witness smiling while describing a distressing event) as well as emotional behaviour (e.g. a witness showing exaggerated calmness when recounting a traumatic experience) can signal dishonesty, while others had the opposite view^1^.

While studies consistently show weak effects and minimal differences between truth-tellers and liars, misconceptions about the diagnostic value of these non-verbal behaviours persist among legal actors and laypersons^5,16–19^. Adding to this complexity, even researchers studying deception hold conflicting views on the universality and consistency of non-verbal cues across situations^20^. However, the relation between non-verbal cues and the accuracy or credibility of eyewitness testimonies remains under-researched, particularly concerning the increased vulnerability of ethnic minorities and those with communication challenges, as noted by Chalmers et al.^15^.

### The present study

Behavioural markers that could differentiate between accurate and inaccurate statements in honestly reported eyewitness testimonies are of both theoretical and practical interest. Identifying such markers holds theoretical importance for understanding the mechanisms by which non-verbal cues influence legal decision-making, as well as practical relevance for addressing potential biases in credibility assessments. To this end, we address the following Research Questions:

i. Are non-verbal cues associated with recall memory accuracy of honestly reported eyewitness statements? We hypothesized that (H1) non-verbal cues will not reliably distinguish between accurate and inaccurate testimony statements, aligning with the deception literature showing weak predictive power of non-verbal cues^3–5^.
ii. Are non-verbal cues associated with witnesses’ self-reported confidence? Consistent with the observation that non-verbal cues can signal a speaker’s own certainty^10^, we hypothesized that (H2) higher self-reported confidence will be associated with distinct non-verbal cues, such as changes in facial expressions (e.g. eyelid tightening or other expressive behaviours as indicated in Table 1 below).
iii. Are non-verbal cues related to observers’ ratings of witness credibility? Based on research showing that jurors and judges often rely on non-verbal cues to assess credibility^1,15^, we hypothesized that (H3) non-verbal cues will be related to credibility ratings by observers. Perceived credibility in the current study refers to the overall quality of a witness’s testimony, including aspects such as their trustworthiness, ability to convey their recollection, and the testimony’s usefulness for the criminal investigation^21^.
iv. Do associations between non-verbal cues, eyewitness accuracy and observers’ credibility judgements differ between native and non-native speaking witnesses? Consistent with H1, we hypothesized (H4a) that non-verbal cues would not be related to accuracy in either language group. However, in line with previous findings (H4b), we expected non-native speakers to display non-verbal cues typically associated with lower perceived credibility, which may have negatively impacted their credibility assessments by independent observers^6,14,15^.

**Table 1:** List of cues included in this study.

Cue	Description
Fidgeting	Directly observable object fidgeting and/or self-fidgeting and/or facial fidgeting
Illustrators	Hand movements that accompany and illustrate speech
Facial shielding	The speaker shielding their face
Shrugs	Up and down movement of the shoulders; and/or the palms of the hand are open, and the hands are moving up and down
Head nods	Affirmative head nods; vertical head movements
Head shakes	Negating head shakes; side-to-side head movements
Other body movement	Movements of the head (including all movements of head except head shakes and head nods), arms, and/or postural adjustments, leanings, trunk movements, and/or repositioning of the body
Smile	The speaker is smiling (even silently); tension lifting corners of their mouth
Eye shifts	Eye movements/shifts in gaze. For example, low gaze (looking downwards) and/or high gaze (looking upwards) and/or side gaze (looking to the left or the right)
Eyes closed	Eyes are closed
Brow movement	Eyebrows are raised or lowered
Other facial expressiveness	The speaker’s face is animated and/or expressive, deviating from a neutral expression (e.g. grimacing, eyelids tightening)

In our study, we focused on 12 non-verbal cues based on the meta-analytic review by DePaulo et al.^3^. Table 1 presents the list of cues and a definition of each one (for cue selection process, see Methods section). The preregistration, materials, and data in this study are available here: https://osf.io/8e2ns/?view_only=61238b73d2ae4f41a831d735dd856f21.

## Methods

### Eyewitness statements

The current dataset was originally published in Raver et al.^6^ and comprises 680 (n_correct_ = 479 and n_incorrect_ = 201) objectively verifiable statements from 36 video-recorded mock eyewitness testimonies. The original study was approved by the Swedish Ethical Review Authority (File number: 2020-00624). Included in the approval of this original study was asking participants for permission to show their testimonies to other participants in subsequent studies. The current study did not include aspects that warrant approval from the Ethical Review Authority (e.g. sensitive personal information about participants) but was conducted in accordance with the general ethical guidelines. Written informed consent was obtained from all participants prior to their participation. Only information relevant for the present study is reported here; for comprehensive details of the materials and procedure used in the original study, see Raver et al.^6^.

Initially, 121 eyewitnesses (50% women; M_age_ = 31.05; SD_age_ = 10.07) first viewed a 36-s muted mock-crime video depicting a violent assault, filmed from an eyewitness point-of-view^22^. After watching the crime, eyewitnesses were interviewed digitally (Zoom) in either their native (Swedish) or non-native (English) language. Non-native eyewitnesses assessed their English proficiency using a shortened version of the Common European Framework of Reference for Languages’ Global Scale^23^, selecting Basic (n = 4), Independent (n = 34), or Proficient (n = 22), with one missing response. Participants also rated their comfort speaking English on a 7-point scale (1 = not at all comfortable, to 7 = very comfortable; *M* = 5.30, *SD* = 0.91, *Mdn* = 6). Participants who reported Proficient and rated their comfort speaking English as 7 were excluded. A free recall task was followed by cued recall using seven open-ended questions (e.g. “*You mentioned a person who got assaulted, can you describe what that person looked like*?”). As eyewitnesses gave their testimonies during the cued recall phase, the interviewer documented details (e.g. ”*perpetrator black jacket*”) on a numbered sheet. After the interview, the interviewer read aloud the details reported by the eyewitness, who, after each detail, rated their confidence in the statement on a scale from 0 to 100% with 20% integers. As the interviewer documented details in real-time, some details could not be captured, hence, not all statements had self-reported confidence ratings. Overall, witnesses were more confident in accurate statements (*M* = 87.55, *SD* = 20.65) than in inaccurate statements (*M* = 80.35, *SD* = 24.06; *d* = -0.33, *p* < 0.001). Native speakers (*M* = 87.10, *SD* = 20.86) were overall more confident than non-native speakers (*M* = 83.49, *SD* = 23.01; *d* = 0.16, *p* = 0.045).

From the initial pool of 121 video-taped testimonies, we screened and selected 36 witnesses as targets. Selection criteria included equal distribution of the two language conditions (native vs. non-native speakers) and gender, good video and audio quality, and testimony accuracy levels reflecting the overall accuracy distribution in the full dataset^6^. For additional details on the screening process, readers are referred to Raver et al.^6^: https://osf.io/zkjrb. Next, native Swedish-speaking raters (N = 202; sample size determined based on preregistered target sample size that were possible within the study’s resources; see sensitivity analysis here: https://osf.io/r3pgn) were recruited and randomly assigned to watch a video of either a native or a non-native speaking eyewitness. Raters rated their English comprehension as high (M = 6.09, SD = 0.97) on a 7-point scale ("*How good do you think you are at understanding English?*"). Each eyewitness was rated by an average of 5–6 raters. Raters were instructed to imagine themselves in the role of an interrogator as they would watch an investigative interview with a person who had witnessed a serious violent crime. After viewing the interview, raters first formulated four questions for a follow-up interview, aimed at obtaining information that could help solve the crime. Then, raters also assessed eyewitnesses’ credibility (1 = not at all; 7 = very much) using eight items ( “*To what extent did you perceive* (a) *the witness’s memory of the event as accurate?,* (b) *that the witness was able to convey their memory of the event?,* (c) *the witness as confident in their statement?,* (d) *the witness as motivated to try to remember the event?*, (e) *the witness as truthful?*,” (f) *to what extent do you assess that the testimony is useful for the criminal investigation?*, (g) *how do you think the witness experienced the situation of being questioned about the event?*, and (h) *how nervous do you perceive the witness was during the interrogation?*; Cronbach’s α = 0.83)^14^. Credibility ratings were averaged across the eight items to create a composite score for each witness. Native speakers were perceived as significantly more credible (*M* = 4.85, *SD* = 0.80) than non-native speakers (*M* = 4.51, *SD* = 1.04; *d* = 0.36, *p* = 0.047)^6^.

### Selection of non-verbal behaviour

We conducted an extensive selection process to identify relevant non-verbal cues, based on definitions from DePaulo et al.^3^. Considering technical limitations of the data (e.g., camera angles, witness visibility, and video quality; see Ref.^24^), we initially identified forty-four potential cues (such as blinking, sneers, specific hand movements etc.). We then revised this list of behaviours through a four-step process to determine the most suitable cues for our study: First, we narrowed the list to seventeen theoretically and empirically relevant cues. Second, we developed a coding manual outlining specific guidelines (see Supplementary materials). Third, the manual was piloted by a research assistant on five randomly selected witnesses (3 native speakers, 2 non-native speakers). Fourth, we refined the cues based on pilot results, merging similar ones – for example, postural shifts and head movements were combined into *Other body movement*, and high gaze and low gaze into *Eye shifts*. This process led to the final selection of twelve non-verbal cues for the study*.*

### Coding of non-verbal behaviour

Statements were selected based on the criteria outlined by Gustafsson et al.^22^, ensuring they could be objectively assessed for veracity (e.g., correct statement = “*uhm like I remember that the one with the beige jacket grabbed the victim*”, accurately describing the person who grabbed the victim; incorrect statement = “*the first, the the one who was talking to the boy who was stabbed, ehm.. Eh, had a black jacket*”, incorrectly describing the brown jacket as black). Before the coding of non-verbal cues, coders underwent thorough training using a specific coding template with instructions and principles to follow to ensure consistency (see Supplementary materials). Coders reviewed both the video recording and the transcription of each selected statement. For each statement, coders noted whether each of the 12 non-verbal cue was either present (1) or absent (0). We used two coders: the first coder coded 17% of the data, and then a second coder independently coded the same 17%. Any disagreements, such as a behaviour being coded as *Fidgeting* by one coder but as *Other body movement* by the other coder, or determining to which statement a particular non-verbal cue belongs in cases where cues would either precede or be slightly delayed in relation to the verbal content, were resolved through discussion (see Table 2 for inter-rater reliability). The second coder then proceeded to code the remaining data. The coders were blind to statement accuracy, perceived credibility by independent observers, and self-reported confidence of each eyewitness.

**Table 2:** Inter-rater reliability for non-verbal cues based on 17% of the eyewitness statements.

Cue	% Agreement	Gwet’s AC1
Fidgeting	99.17	0.93
Illustrators	100	1
Facial shielding	100	1
Shrugs	100	1
Head nods	99.17	0.88
Head shakes	100	1
Other body movement	86.78	0.59
Smile	99.17	0.80
Eye shifts	91.74	0.81
Eyes closed	98.35	0.88
Brow movement	95.87	0.90
Other facial expressiveness	98.35	0.93

% Agreement shows the proportion of instances where both coders gave identical codes, indicating simple agreement without adjusting for chance. Gwet’s AC1 values range from − 1 to 1, where 1 indicates perfect agreement, 0 indicates no agreement beyond chance, and negative values indicate agreement worse than what would be expected by chance^25^.

### Data analysis

The coded statements in the current study included statements from both the free recall and cued recall phases. Non-verbal cues and accuracy were coded at the statement level across both recall phases, while self-reported confidence was measured at the statement level during only the cued recall phase. Credibility, in contrast, was assessed as an aggregated witness-level mean score based solely on the free recall phase. See Limitations section for a discussion on the different bases for the analyses. In our generalized linear mixed-effects models (GLMM), we fitted separate models for each behavioural cue to investigate the likelihood of the cue being exhibited based on accuracy (correct vs. incorrect), witness language (native vs. non-native), perceived credibility by independent observers (1 = not at all; 7 = very much) and the witness’ self-reported confidence in statements (from 0 to 100% with 20% integers). We added interaction effects between accuracy and language, as well as between credibility and language, to examine whether the effects of accuracy and perceived credibility varied between native and non-native speakers. Also, since witnesses may differ in their tendency to exhibit certain cues, we included a random intercept for individual witnesses to account for individual differences. We then compared baseline null-models with the intercept of cue and random intercept for witness only; against models adding our predictors as fixed effects. For our model comparisons, we used Akaike weights (values ranging from 0 to 1, where larger values indicate stronger evidence compared to other models being considered; see Ref.^26^. With a Bonferroni correction for testing 12 cues, the alpha level was adjusted to 0.004 to determine statistical significance. All statistical analyses were conducted using R through RStudio^27,28^.

## Results

In the results section, we first present the frequency of non-verbal cues observed in correct versus incorrect statements, across native versus non-native language speakers (see Table 3). We then detail the findings from the GLMMs, examining the effects of accuracy, language, and their interaction on the likelihood of non-verbal cues being exhibited by a witness (see Table 4). Next, we examine the effects of witness’ perceived credibility on the likelihood of non-verbal cues being exhibited, and if it interacts with witness’ language (see Table 5). For these analyses, *Facial Shielding* and *Eyes Closed* were omitted due to model outputs yielding ORs of 0 or infinity, leading to unreliable estimates. Finally, considering that not all witness statements included self-reported confidence judgments, we conducted a set of separate complete case models for statements with available confidence data to investigate the relation between self-reported confidence and non-verbal cues (see Table 6). Random effects variance across witnesses, considering all 12 non-verbal cues, ranged from 0.59 to 3.70 (*M* = 1.42; *SD* = 0.97; *Mdn* = 1.09), indicating that some witnesses exhibited more non-verbal cues overall than others. A detailed report of all statistical analyses, including the code and model output, can be found here: https://osf.io/vpe7k?view_only=61238b73d2ae4f41a831d735dd856f21.

**Table 3:** Absolute and relative frequencies of non-verbal cues exhibited by native and non-native speakers in correct and incorrect statements.

Cue	Overall	Correct statements (n = 479)			Incorrect statements (n = 201)		
		Total	Native	Non-native	Total	Native	Non-native
Overall cues^1^	1632	1136	567	569	496	288	206
Fidgeting	60 (8.82%)	44 (9.19%)	26 (10.74%)	18 (7.59%)	16 (7.96%)	12 (10.17%)	4 (4.88%)
Illustrators	78 (11.47%)	59 (12.32%)	37 (15.29%)	22 (9.28%)	19 (9.45%)	9 (7.63%)	10 (12.2%)
Facial shielding	5 (0.74%)	3 (0.63%)	3 (1.24%)	0 (0%)	2 (1%)	2 (1.69%)	0 (0%)
Shrugs	11 (1.62%)	7 (1.46%)	6 (2.48%)	1 (0.42%)	4 (1.99%)	3 (2.54%)	1 (1.22%)
Head nods	165 (24.26%)	114 (23.8%)	54 (22.31%)	60 (25.32%)	51 (25.37%)	32 (27.12%)	19 (23.17%)
Head shakes	93 (13.68%)	58 (12.11%)	30 (12.4%)	28 (11.81%)	35 (17.41%)	22 (18.64%)	12 (14.63%)
Other body movement	421 (61.91%)	299 (62.42%)	154 (63.64%)	145 (61.18%)	122 (60.7%)	70 (59.32%)	52 (63.41%)
Smile	19 (2.79%)	14 (2.92%)	6 (2.48%)	8 (3.38%)	5 (2.49%)	3 (2.54%)	2 (2.44%)
Eye shifts	436 (64.12%)	296 (61.8%)	137 (56.61%)	159 (67.09%)	140 (69.65%)	81 (68.64%)	58 (70.73%)
Eyes closed	41 (6.03%)	32 (6.68%)	22 (9.09%)	10 (4.22%)	9 (4.48%)	9 (7.63%)	0 (0%)
Brow movement	195 (28.68%)	138 (28.81%)	65 (26.86%)	73 (30.8%)	57 (28.36%)	31 (26.27%)	26 (31.71%)
Other facial expressiveness	108 (15.88%)	72 (15.03%)	27 (11.16%)	45 (18.99%)	36 (17.91%)	14 (11.86%)	22 (26.83%)

Percentages represent the proportion of observed instances of each non-verbal cue relative to the total number of statements within that category. Although the total count of exhibited cues is higher in correct (vs. incorrect) statements, this is due to the larger number of correct (vs. incorrect) statements in the dataset. ^1^Refers to the sum of all observed non-verbal cues.

**Table 4:** Generalized mixed-effects model predicting non-verbal cues based on Accuracy (correct vs. incorrect, with correct as the reference group), Language (native vs. non-native, with native as the reference group), and their interaction.

Cue	Predictor					
	Accuracy incorrect		Non-native language		Accuracy incorrect x non-native language	
	OR [95% CI]	*p*	OR [95% CI]	*p*	OR [95% CI]	*p*
Overall cues	1.03 [0.89, 1.19]	0.674	1.03 [0.83, 1.26]	0.813	1.03 [0.83, 1.28]	0.764
Fidgeting	1.02 [0.43, 2.44]	0.957	0.78 [0.12, 4.89]	0.792	0.61 [0.14, 2.73]	0.521
Illustrators	0.32 [0.13, 0.75]	0.009	0.34 [0.08, 1.53]	0.162	4.02 [1.17, 13.87]	0.027
Shrugs	0.90 [0.20, 4.15]	0.897	0.48 [0.02, 13.34]	0.666	3.31 [0.13, 81.87]	0.463
Head nods	1.58 [0.90, 2.78]	0.114	1.61 [0.65, 3.99]	0.301	0.64 [0.27, 1.51]	0.309
Head shakes	1.81 [0.90, 3.64]	0.097	1.48 [0.50, 4.38]	0.474	0.91 [0.32, 2.62]	0.862
Other body movement	0.77 [0.47, 1.26]	0.292	0.93 [0.45, 1.90]	0.834	1.41 [0.66, 2.99]	0.371
Smile	1.29 [0.29, 5.63]	0.739	1.31 [0.21, 8.00]	0.773	0.65 [0.07, 5.80]	0.699
Eye shifts	1.47 [0.88, 2.47]	0.139	1.33 [0.54, 3.25]	0.531	0.93 [0.41, 2.08]	0.851
Brow movement	0.94 [0.54, 1.63]	0.823	1.12 [0.43, 2.90]	0.819	1.11 [0.49, 2.52]	0.809
Other facial expressiveness	1.09 [0.54, 2.20]	0.821	2.15 [0.93, 5.01]	0.075	1.36 [0.53, 3.51]	0.523

The p-values are raw scores, with the Bonferroni corrected significance threshold of *p* = 0.004. Number of observations = 679. *OR* = Odds Ratio; CI = confidence interval. Odds Ratio measures the strength of association: OR > 1 indicates greater odds, OR = 1 indicates no difference, and OR < 1 indicates lower odds.

**Table 5:** Generalized mixed-effects model predicting non-verbal cues based on credibility (z-transformed) and its interaction with Language (native vs. non-native, with native as the reference group).

Cue	Predictor			
	Credibility		Credibility x non-native language	
	OR [95% CI]	*p*	OR [95% CI]	*p*
Overall cues	1.03 [0.90, 1.19]	0.639	0.99 [0.82, 1.19]	0.920
Fidgeting	1.53 [0.42, 5.62]	0.524	0.39 [0.07, 2.08]	0.267
Illustrators	0.93 [0.33, 2.65]	0.898	0.67 [0.17, 2.65]	0.567
Shrugs	6.15 [1.54, 24.59]	0.010	0.07 [0.01, 0.54]	0.010
Head nods	1.71 [0.92, 3.16]	0.087	0.63 [0.28, 1.43]	0.266
Head shakes	2.49 [1.23, 5.05]	0.011	0.45 [0.17, 1.16]	0.098
Other body movement	0.84 [0.52, 1.37]	0.483	1.77 [0.92, 3.39]	0.088
Smile	1.20 [0.34, 4.21]	0.774	0.47 [0.09, 2.35]	0.356
Eye shifts	0.94 [0.51, 1.74]	0.842	1.15 [0.51, 2.61]	0.736
Brow movement	0.76 [0.39, 1.46]	0.407	1.41 [0.59, 3.37]	0.436
Other facial expressiveness	1.11 [0.62, 1.98]	0.731	0.92 [0.44, 1.93]	0.824

The p-values are raw scores, with the Bonferroni corrected significance threshold of *p* = 0.004. Number of observations = 679. *OR* odds Ratio; *CI* confidence interval. Odds Ratio measures the strength of association: OR > 1 indicates greater odds, OR = 1 indicates no difference, and OR < 1 indicates lower odds.

**Table 6:** Generalized mixed-effects model predicting non-verbal cues based on witnesses’ self-reported confidence in their statements.

Cue	OR [95% CI]	*p*
Fidgeting	0.99 [0.97, 1.01]	0.173
Illustrators	1.02 [1.00, 1.04]	0.086
Facial shielding	0.96 [0.90, 1.02]	0.199
Shrugs	0.99 [0.93, 1.04]	0.616
Head nods	1.00 [0.99, 1.02]	0.600
Head shakes	0.99 [0.97, 1.00]	0.068
Other body movement	0.99 [0.98, 1.01]	0.319
Smile	0.99 [0.96, 1.03]	0.655
Eye shifts	1.00 [0.99, 1.01]	0.731
Eyes closed	0.99 [0.97, 1.01]	0.445
Brow movement	0.99 [0.98, 1.00]	0.171
Other facial expressiveness	0.97 [0.96, 0.98]	** < 0.001**

Statistically significant p-values are boldfaced. The p-values are raw scores, with the Bonferroni corrected significance threshold of *p* = 0.004. Number of observations = 455. *OR* odds ratio; *CI* confidence interval. Odds Ratio measures the strength of association: OR > 1 indicates greater odds, OR = 1 indicates no difference, and OR < 1 indicates lower odds.

For both correct and incorrect statements, the most frequently observed non-verbal cues were *Other body movement*, *Eye shifts*, *Head nods*, and *Brow movement*, with *Facial shielding*, *Shrugs*, *Eyes closed*, and *Smile* being the least frequent (see Table 3). This pattern was consistent across native and non-native speakers. However, some notable differences in observed frequencies emerged. Native speakers used *Illustrators* more in correct (15.29%) than in incorrect statements (7.63%), whereas non-native speakers tended to use more in *Illustrators* during incorrect statements (10.2%) compared to correct ones (9.28%). Non-native speakers displayed *Other Facial Expressiveness* more frequently in incorrect (26.83%) than correct statements (18.99%). Native speakers displayed both *Head Nods* (27.12% vs. 22.31%) and *Head Shakes* (18.64% vs. 12.4%) more frequently in incorrect statements compared to correct ones. Despite the higher absolute number of cues observed in correct statements compared to incorrect ones, the actual mean number of non-verbal cues was approximately 2.4 cues per correct statement (1136 cues/479 statements) compared to 2.5 cues per incorrect statement (496 cues/201 statements). Notably, native speakers exhibited more cues overall in incorrect statements (n = 288) compared to non-native speakers (n = 206), a pattern not observed in correct statements (567 vs. 569, respectively).

### Non-verbal cues, accuracy and witness language

The results of the GLMMs, with the adjusted Bonferroni-corrected alpha threshold of 0.004, revealed no statistically significant associations between any of the non-verbal cues and accuracy (correct vs. incorrect), language (native vs. non-native), or their interaction (see Table 4). While none of the cues reached statistical significance, some demonstrated notable trends. For instance, the use of *Illustrators* was approximately three times less likely in incorrect statements compared to correct ones (OR = 0.32, 95% CI [0.13, 0.75]). Non-native speakers were four times more likely to use *Illustrators* in incorrect statements compared to correct ones (OR = 4.02, 95% CI [1.17, 13.87]). Additionally, *Head Shakes* were nearly twice as likely to be observed in incorrect statements than in correct ones (OR = 1.81, 95% CI [0.90, 3.64]), and *Other Facial Expressiveness* occurring more than twice as often in non-native compared to native speakers (OR = 2.15, 95% CI [0.93, 5.01]). Also, total number of cues was not associated with accuracy (OR = 1.03, 95% CI [0.89, 1.19]), and we found no differences between native and non-native speakers in the overall number of cues (OR = 1.03, 95% CI [0.83, 1.26]).

### Non-verbal cues, perceived credibility and witness language

We found no significant associations between non-verbal cues and credibility, or between credibility and its interaction with language (native vs. non-native; see Table 5). However, *Shrugs* were observed over six times more often in statements with higher perceived credibility (OR = 6.15, 95% CI [1.54, 24.59]). The interaction between credibility and language indicated that non-native speakers were approximately 14.3 times less likely to exhibit *Shrugs* with higher perceived credibility (OR = 0.07, 95% CI [0.01, 0.54), compared to their native speaking counterparts. Additionally, *Head Shakes* were more than twice as likely to be observed with higher perceived credibility (OR = 2.49, 95% CI [1.23, 5.05]), and *Eyes Closed* was approximately five times less likely to occur with higher perceived credibility (OR = 0.21, 95% CI [0.04, 1.09]); yet these remained non-significant. Again, witnesses’ overall non-verbal cues were not associated with perceived credibility levels (OR = 1.03, 95% CI [0.90, 1.19]), and no interaction was found between native and non-native speakers in exhibited overall cues (OR = 0.99, 95% CI [0.82, 1.19]).

Mirroring the analyses above, we conducted separate follow-up analyses for each credibility item and found consistent results: no statistically significant associations between non-verbal cues and any individual credibility item. The ORs ranged from 0.99 to 1.18 (*M* = 1, *SD* = 0.02, *Mdn* = 1), with p-values ranging from 0.589 to 0.998 (*M* = 0.95, *SD* = 0.06, *Mdn* = 0.97). See detailed report here: https://osf.io/vpe7k?view_only=61238b73d2ae4f41a831d735dd856f21.

### Self-reported confidence

In terms of the relation between non-verbal cues and witnesses’ self-rated confidence (see Table 6), *Other Facial Expressiveness* was the only cue that reached the adjusted statistical significance threshold. Specifically, lower self-reported confidence in statements was associated with a higher likelihood of displaying *Other Facial Expressiveness* (OR = 0.97). However, the effect size was minimal, with only a 3% decreased likelihood, suggesting that the relation may not be practically significant.

No significant associations with self-reported confidence were found for *Fidgeting*, *Illustrators*, *Facial Shielding*, *Shrugs*, *Head Nods*, *Head Shakes*, *Other Body Movement*, *Smile*, *Eye Shifts*, *Eyes Closed*, or *Brow Movement*.

For *Shrugs* but for no other non-verbal cue did the model with predictors (accuracy, language, perceived credibility, and their interactions) show a better fit than the baseline null-model, *w*_*i*_(AIC) = 0.59*.* For all other cues, the baseline null-models had a better fit, with the Akaike weights for these models ranging between 0.01 and 0.48. This suggests that the predictors did not significantly improve model fit for these cues.

## Discussion

In this study, we explored several key aspects of non-verbal cues in eyewitness testimonies. First, we examined whether non-verbal cues were associated with testimony statements’ accuracy (RQ1). We found support for (H1) that non-verbal cues would not reliably distinguish accurate from inaccurate witness statements. We then investigated the relation between non-verbal cues and witnesses’ self-reported confidence (RQ2). We found partial support of (H2) showing that higher self-reported confidence would be associated with distinct non-verbal cues. Additionally, we explored whether non-verbal cues were related to ratings of witness credibility by independent observers (RQ3). Here, we found no support of (H3) showing that non-verbal cues would influence observers’ credibility ratings. Finally, we examined whether these associations differed between native and non-native speaking witnesses (RQ4). We found support of (H4a) showing that non-verbal cues would not be related to accuracy in either language group, and (H4b) no support that non-native speakers would display cues commonly associated with uncertainty, potentially affecting their perceived credibility.

### Non-verbal cues and accuracy in native vs. non-native eyewitness testimonies

Our findings contradict common conceptions that certain behaviours signal the accuracy of a statement. Behaviours such as *Fidgeting*, *Facial shielding*, or *Eye Shifts* – often signs of nervousness or dishonesty – did not reliably distinguish correct statements from incorrect ones, aligning with the deception literature showing weak predictive power of non-verbal cues^3–5^. Thus, our findings attest to the notion that there are no clear non-verbal markers that allow people to determine whether a statement is accurate or not. Hence, relying on a witness’ non-verbal behaviour to assess the accuracy of a statement is likely to result in misjudgements, with potential far-reaching consequences in legal contexts. Furthermore, non-native speakers did not exhibit distinct non-verbal cues that could be linked to the accuracy of their statements. This, again, underscores the risk of misinterpreting non-verbal cues when assessing statement accuracy with native and non-native speakers^15^.

### Non-verbal cues and their relation to witnesses’ self-reported confidence

Our analysis revealed a significant association between *Other Facial Expressiveness* (e.g., animated facial expressions that deviate from neutral, such as grimacing or eyelid tightening) and lower self-reported witness’ confidence in statements. This finding aligns clearly with previous research showing that non-verbal behaviours can reflect a speaker’s Feeling of Knowing^10^. However, the small effect sizes observed suggest that the relation between facial expressions and self-reported confidence may not be robust and warrants further investigation. Also, no significant associations were found between self-reported confidence and other non-verbal cues. Hence, the usefulness of non-verbal cues to estimate witnesses’ own confidence in practical contexts seems limited.

### Non-verbal cues and credibility assessment of native vs. non-native speakers

Research by Chalmers et al.^15^ showed that jurors frequently report relying on cues such as body language and gaze direction to judge credibility, despite the lack of evidence supporting the reliability of these indicators. Similarly, Denault et al.^1^ found that judges often claim to base their credibility assessments on a witness’s gestures, gaze, and posture, believing these cues reflect truthfulness. However, we find no association between witnesses’ non-verbal behaviour and their perceived credibility. Thus, jurors may erroneously believe both that non-verbal cues can reliably differentiate between accurate and inaccurate statements, and that they themselves use these cues when assessing witness credibility, even though the evidence supporting such assumptions is context-dependent and limited. For example, studies like McKimmie et al.^29^ have shown that non-verbal behaviours can influence jurors’ perceptions under specific conditions, such as when stereotypically non-deceptive cues are accessible. These findings highlight the variability in observers’ interpretations of non-verbal cues, which may depend on prior expectations and the salience of such cues in legal context. We had expected that the lower credibility ascribed to non-native vs. native witnesses^6^ would be due to non-natives displaying more non-verbal cues associated with uncertainty. However, native and non-native speakers did not differ in their use of non-verbal cues, hence the harsher judgments of non-native speakers found in previous research^6,14^ are likely influenced by other factors. Further research should thoroughly explore the basis for this difference in perceived credibility, considering factors such as ethnic discrimination or cultural biases^15^.

### Limitations

First, considering technical limitations (e.g. camera angles, witness visibility, and video quality; see Ref.^24^, the video-taped eyewitness testimonies used in the current study may not fully represent the diverse range of non-verbal behaviours observed in real-world settings. This limitation is particularly pertinent given that our initial process identified 44 potential cues, which were subsequently reduced to the 12 cues we deemed most suitable for our study aims. Second, inter-rater reliability for one of the cues, *Other body movement,* was lower than desired (Gwet’s AC1 = 0.59), indicating only moderate agreement despite a high percentage of overall agreement (86.78%). This was the case since coders often identified the cue, but their interpretations were not always consistent. This clearly attest to the difficulty and subjectivity involved in observing and interpreting non-verbal cues – a challenge that extends beyond research to real-world contexts. In legal settings, such subjectivity can lead to different interpretations of the same behaviour, potentially resulting in inconsistent and biased judgements, influenced by the observer’s expectations or cultural background. Additionally, the overlap in inter-rater coding was limited to 17% (122 statements) due to time and resource constraints, which, while not ideal, provided a sufficiently large sample to ensure reliability. Third, the relatively small sample of 36 unique witnesses (18 native, 18 non-native) and the single-judgment-per-observer design, with each witness rated approximately 5–6 times, may limit the generalizability of the findings^30^. Future research should address this by including more witnesses and increasing the number of judgments per observer. Fourth, the different bases for the analyses for non-verbal cues, accuracy, credibility assessments, and self-reported confidence introduce additional limitations, in that, it may impede the interpretability of the findings. For the Raver et al.^6^ study, witnesses’ self-reported confidence was measured during only the cued recall phase and was used to examine the confidence-accuracy relation; hence, self-reported confidence data was analysed separately for the cued recall phase. Credibility assessments were measured only in the free recall phase to minimize interviewer influence (see Raver et al.^6^) but were related to both free and cued recall data in the current study in order to avoid significantly reducing the available dataset. Hence, it is important to note that the credibility measure reflect an overall assessment based solely on free recall and therefore does not take into account the testimony provided during cued recall. However, follow-up analyses in the current study, using only the free recall phase, demonstrated results consistent with the original analyses, further supporting the robustness of our findings (see: https://osf.io/6cqsm). Future research should, however, align the bases for the analyses to enhance consistency and interpretability. Despite these limitations, this study represents a state-of-the-art contribution to understanding the relation between non-verbal cues, accuracy, credibility assessments, and self-reported confidence in honestly reported eyewitness testimonies. By examining these associations, particularly in the context of native and non-native speakers, this research provides valuable insights that advance both theoretical understanding and practical applications in legal contexts.

## Conclusions

The current study highlights the challenges of using non-verbal cues in legal contexts to inform credibility assessments and decision-making. Although some trends were observed between specific non-verbal cues and predictors – such as the threefold decreased likelihood of using Illustrators in incorrect statements compared to correct ones, and Shrugs being observed over six times more often in statements with higher perceived credibility – the overall effect sizes were minimal. Importantly, our findings showed no significant differences in exhibited non-verbal cues between native and non-native speakers. Taken together, we demonstrate minimal predictive value of non-verbal cues for determining testimony accuracy, perceived credibility, or self-reported confidence. This contrasts clearly with the high reliance on such cues in real-world legal contexts. Although non-verbal cues may be more pronounced in real-life high-stakes forensic settings, the variability across individuals and lack of predictive value for testimony accuracy caution against relying on them for credibility assessments. In other words, these cues are simply not helpful when trying to figure out the accuracy of witnesses. In sum, this study underscores the need for a cautious and evidence-based approach when interpreting non-verbal cues, particularly in high-stakes legal settings.

## Supplementary Information

Supplementary Information 1.

Supplementary Information 2.

## Data Availability

The data that support the findings of this study are openly available in https://osf.io/8e2ns/?view_only=61238b73d2ae4f41a831d735dd856f21.
