# Supplementary material for: Non-verbal cues in eyewitness testimonies do not predict accuracy or credibility assessments
Source: Sci Rep. 2025 Feb 12;15:5265. doi: 10.1038/s41598-025-89825-0 (PMC11821912; doi:10.1038/s41598-025-89825-0)
Supplement: Supplementary file 1 — Supplementary Information 1. [file 41598_2025_89825_MOESM1_ESM.docx]

**NON-VERBAL CUES IN EYEWITNESS TESTIMONIES DO NOT PREDICT ACCURACY OR CREDIBILITY ASSESSMENTS**

Arman Raver*, Torun Lindholm, and Charlotte Alm

Department of Psychology, Stockholm University

**Supplementary Figures**

**Figure S1.** Generalized mixed-effects model predicting non-verbal cues based on Accuracy (correct vs. incorrect, with correct as the reference group), Language (native vs. non-native, with native as the reference group), and their interaction.


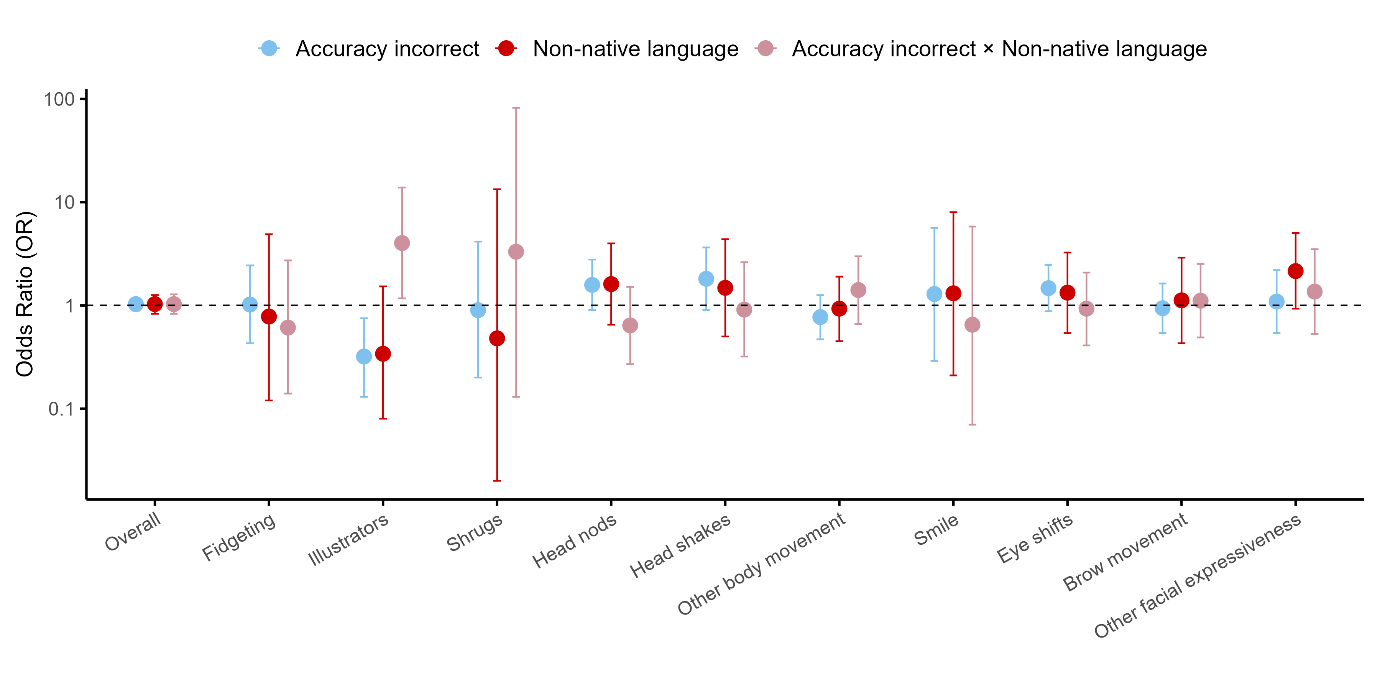


*Note*. Each point represents the OR for a specific non-verbal cue, with 95% confidence intervals (CI) depicted by error bars. The dashed line at OR = 1 represents no effect. An OR greater than 1 indicates increased likelihood of the cue being observed, while an OR less than 1 indicates decreased likelihood. CIs that include 1 indicates that the likelihood of observing a non-verbal cue is as likely as not observing the cue, suggesting that the observed effect is not statistically significant. In sum, the ORs ranged from 0.32 to 4.02 (*M* = 1.24; *SD* = 0.75; *Mdn* = 1.03), with *p*-values ranging from .009 to .998 (*M* = .585; *SD* = 0.3; *Mdn* = .674).

**Figure S2**. Generalized mixed-effects model predicting non-verbal cues based on Credibility (z-transformed) and its interaction with Language (native vs. non-native, with native as the reference group).


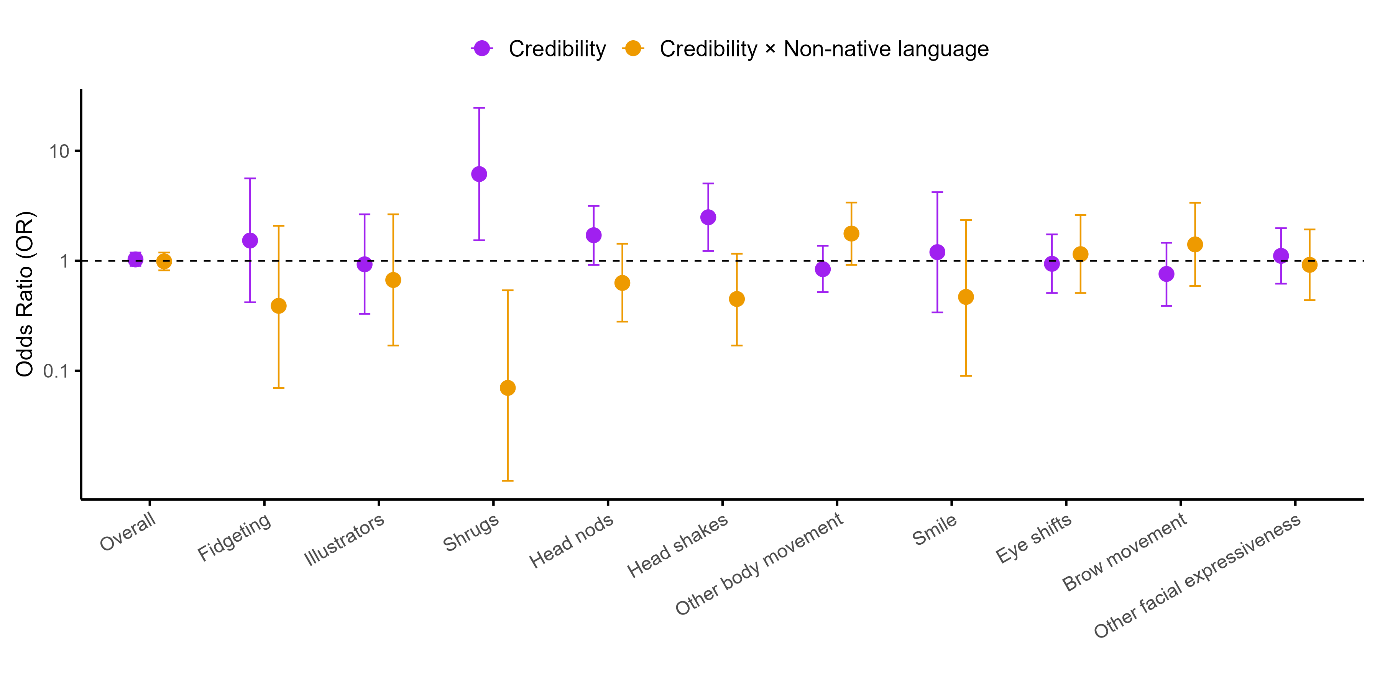


*Note*. Each point represents the OR for a specific non-verbal cue, with 95% CIs depicted by error bars. The dashed line at OR = 1 represents no effect. An OR greater than 1 indicates increased likelihood of the cue being observed, while an OR less than 1 indicates decreased likelihood. CIs that include 1 indicates that the likelihood of observing a non-verbal cue is as likely as not observing the cue, suggesting that the observed effect is not statistically significant. In sum, the ORs ranged from 0.07 to 6.15 (*M* = 1.26; *SD* = 1.22; *Mdn* = 0.96), with *p*-values ranging from .010 to .920 (*M* = .451; *SD* = 0.32; *Mdn* = .459).

**Figure S3**. Generalized mixed-effects model predicting non-verbal cues based on Confidence (z-transformed).


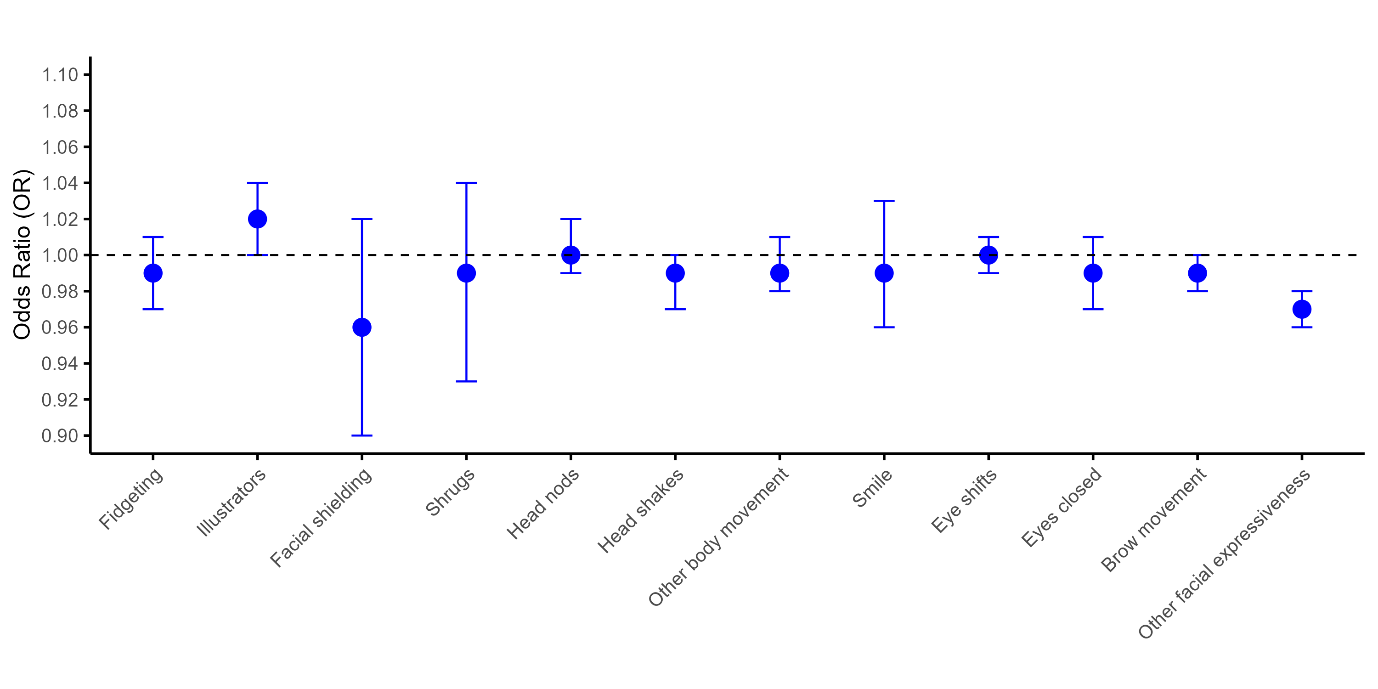


*Note*. Each point represents the OR for a specific non-verbal cue, with 95% CIs depicted by error bars. The dashed line at OR = 1 represents no effect. An OR greater than 1 indicates increased likelihood of the cue being observed, while an OR less than 1 indicates decreased likelihood. CIs that include 1 indicates that the likelihood of observing a non-verbal cue is as likely as not observing the cue, suggesting that the observed effect is not statistically significant. In sum, the ORs ranged from 0.96 to 1.02 (*M* = 0.99; *SD* = 0.02; *Mdn* = 0.99), with p-values ranging from <.001 to .731 (*M* = 0.356; *SD* = 0.236; *Mdn* = 0.319).
