# Supplementary material for: Non-verbal cues in eyewitness testimonies do not predict accuracy or credibility assessments
Source: Sci Rep. 2025 Feb 12;15:5265. doi: 10.1038/s41598-025-89825-0 (PMC11821912; doi:10.1038/s41598-025-89825-0)
Supplement: Supplementary file 2 — Supplementary Information 2. [file 41598_2025_89825_MOESM2_ESM.docx]

**NON-VERBAL CUES IN EYEWITNESS TESTIMONIES DO NOT PREDICT ACCURACY OR CREDIBILITY ASSESSMENTS**

Arman Raver*, Torun Lindholm, and Charlotte Alm

Department of Psychology, Stockholm University

**Supplementary Materials**

**Instructions coding non-verbal cues**

**Procedure**

1. For each statement, each non-verbal cue should be coded as either present (1) or absent (0).

**Principles**

- Reserve coding (1 = present vs. 0 = absent) for clear expressions of non-verbal cues.
  - In cases where it is unclear (e.g., if you need to view the same statement for the same non-verbal cue multiple times): code as present (1) but document these cases (e.g., highlight the cell) for discussion.
  - In cases where the intensity varies within the same witness (e.g., subtle movements of a specific non-verbal cue followed by exaggerated expressions of the same non-verbal cue that question previous assessments): code these as present (1) but document these cases (e.g., highlight the cell) for discussion.
- Each non-verbal cue is coded independently and considered separately from other cues. For example, each *Smile* and/or *Brow movement* is not automatically part of *Other facial expressiveness*; each *Fidgeting*, *Illustrator*, *Facial* *shielding*, and/or *Shrugs* is not automatically part of *Other* *body* *movement*.
  - Code present (1) for each specific non-verbal cue observed.
  - If it is unclear which category a certain cue belongs to (e.g., whether it is a *Brow* *movement* or *Other facial* *expressiveness*): document these cases (e.g., highlight the statement and the cue) for further discussion.
- A statement begins and ends with the first and last word indicated in the Data file – only code for the non-verbal cues expressed within each statement.
  - Non-verbal cues expressed just before and/or just after a statement should not be coded.
  - When statements come directly after one another, be careful to identify and code non-verbal cues for each specific statement separately. This means you need to be attentive to which statement a certain non-verbal cue belongs to.
    - If it is difficult to determine which statement a non-verbal cue belongs to, document these cases (e.g., highlight the statement and the cue) for further discussion.
- In cases where image quality and/or camera angle affect the interpretation of non-verbal cues (e.g., unclear if the witness has a downward gaze/looking down or just looking at the camera; unclear if the witness makes a facial expression due to camera angle): document these cases (e.g., highlight the statement and the cue) for discussion.
